# Galectin-1 in Cardiovascular Pathogenesis: Unraveling Dual Roles and Mechanistic Insights in Emerging Research

**DOI:** 10.3390/medicina61061020

**Published:** 2025-05-30

**Authors:** Po-Yuan Chen, Chun-Yao Cheng, Chun-Chao Chen, Huan-Yuan Chen, Ju-Chi Liu, Wen-Rui Hao, Tzu-Hurng Cheng, Jin-Jer Chen

**Affiliations:** 1 Department of Biological Science and Technology, College of Life Sciences, China Medical University, Taichung City 406040, Taiwan; pychen@mail.cmu.edu.tw; 2 Department of Ophthalmology, Cathay General Hospital, Taipei City 10633, Taiwan; b05401143@ntu.edu.tw; 3 Division of Cardiology, Department of Internal Medicine, Shuang Ho Hospital, Ministry of Health and Welfare, Taipei Medical University, New Taipei City 23561, Taiwan; b101092035@tmu.edu.tw (C.-C.C.); liumdcv@tmu.edu.tw (J.-C.L.); 4 Division of Cardiology, Department of Internal Medicine, School of Medicine, College of Medicine, Taipei Medical University, Taipei City 11002, Taiwan; 5 Institute of Biomedical Sciences, Academia Sinica, Taipei City 11529, Taiwan; hchen9@ibms.sinica.edu.tw (H.-Y.C.); jc8510@yahoo.com (J.-J.C.); 6 Department of Biochemistry, School of Medicine, College of Medicine, China Medical University, Taichung City 404328, Taiwan; 7 Division of Cardiology, Department of Internal Medicine, China Medical University Hospital, Taichung 40447, Taiwan

**Keywords:** galectin-1, cardiovascular diseases, dual role, risk assessment, therapeutic target

## Abstract

Galectin-1 (Gal-1), a β-galactoside-binding lectin, plays a complex role in cardiovascular diseases (CVDs), exerting both protective and pathological effects depending on the context. This review synthesizes findings from the past decade to explore Gal-1’s involvement in key aspects of CVD pathogenesis, including vascular homeostasis, inflammation regulation, atherosclerosis progression, myocardial remodeling, and heart failure. While Gal-1 supports endothelial integrity and immune modulation, its dysregulation contributes to disease progression through pro-inflammatory signaling, fibrosis, and adverse cardiac remodeling. Emerging evidence suggests that Gal-1 holds potential as both a biomarker for risk assessment and a therapeutic target. However, critical knowledge gaps remain, particularly regarding its context-dependent effects, the limited scope of clinical trials, and unresolved mechanistic insights. Addressing these challenges will be essential to fully harness Gal-1’s therapeutic potential in cardiovascular medicine, guiding future research efforts toward precision interventions and clinical applications.

## 1. Introduction

Galectins are a family of β-galactoside-binding proteins involved in various aspects of cardiovascular physiology and pathology. Among them, galectin-1 (Gal-1) plays a significant role in immune regulation, inflammation, and vascular remodeling, making it a key factor in cardiovascular disease (CVD) pathogenesis [1]. While extensively studied for its involvement in immune modulation, fibrosis, and endothelial function, Gal-1 is not the only galectin contributing to disease progression. Galectin-3 (Gal-3) and galectin-9 (Gal-9) have also been implicated in CVD, each influencing inflammatory and fibrotic pathways in distinct ways [2]. Gal-3 has been associated with chronic inflammation and myocardial fibrosis, with elevated levels correlating with worse prognosis in heart failure patients [3]. It facilitates monocyte recruitment to the arterial wall, promoting atherosclerosis and plaque instability, thereby increasing the risk of acute coronary syndromes [4]. Moreover, Gal-3 enhances fibroblast activation and collagen deposition, leading to ventricular stiffening and maladaptive cardiac remodeling [2]. Gal-9 plays a complex role in CVD, modulating immune responses through T-cell activation and macrophage polarization [5]. While it exhibits anti-inflammatory effects, excessive Gal-9 expression may contribute to immune dysregulation and endothelial dysfunction, complicating disease progression [5]. Together, these galectins form an intricate regulatory network influencing cardiovascular pathology. Gal-1’s dual role—exhibiting both protective and pathological effects—reflects its context-dependent nature. It supports angiogenesis and endothelial integrity while also contributing to vascular inflammation, atherosclerosis progression, and fibrosis, ultimately affecting cardiac remodeling [1,6]. This complexity underscores the challenges and potential of targeting Gal-1 therapeutically. CVD remains a leading global health burden, driven by metabolic risk factors such as hypertension, dyslipidemia, and diabetes [7]. While preventive strategies aim to mitigate these conditions, understanding molecular contributors like Gal-1 may provide new therapeutic avenues. Its involvement in endothelial dysfunction and immune modulation suggests broader systemic effects beyond localized vascular pathology [8]. Additionally, Gal-1’s interactions with calcium channels and immune cells further influence cardiovascular homeostasis, yet its precise mechanisms require further exploration [5,9]. Recent studies propose Gal-1 as a potential biomarker for disease progression, particularly in metabolic disorders like type 2 diabetes, where its levels fluctuate with disease severity [10]. However, uncertainties remain regarding its systemic impact on cardiovascular outcomes, emphasizing the need for further research into its regulatory mechanisms. This review consolidates current insights into Gal-1’s role in CVDs, highlighting key knowledge gaps and future directions for therapeutic development.

This paper provides a comprehensive analysis of Gal-1 and its role in CVD pathogenesis. By synthesizing recent research, it critically evaluates Gal-1’s involvement in immune regulation, vascular remodeling, and disease progression. Furthermore, emerging evidence suggests Gal-1’s potential as a biomarker and therapeutic target, highlighting both opportunities and limitations in clinical applications. To ensure a rigorous and unbiased review, a systematic search was conducted across multiple academic databases, including PubMed, Scopus, and Web of Science, focusing on peer-reviewed articles published within the past decade. The search strategy incorporated relevant keywords, such as “Galectin-1”, “cardiovascular diseases”, “inflammation”, and “biomarkers”, to identify studies with mechanistic insights and clinical relevance. Reference lists of key publications were also reviewed to capture influential studies that may have been overlooked. The inclusion criteria prioritized research that significantly contributed to the understanding of Gal-1’s role in CVDs, while the exclusion criteria filtered out non-peer-reviewed articles, conference abstracts, and studies with insufficient methodological rigor. Two independent reviewers assessed the relevance and quality of each study, resolving any discrepancies through discussion to enhance reliability. Beyond consolidating existing knowledge, this paper highlights unresolved questions, identifies gaps in the literature, and outlines future research directions to deepen our understanding of Gal-1’s significance in cardiovascular health and disease. By addressing these areas, this review aims to provide valuable insights that can inform both basic research and translational efforts in cardiovascular medicine.

## 2. Galectin-1: A Multifaceted Player in Cardiovascular Health and Disease

### 2.1. Overview of Galectin-1 Structure and Function

Gal-1 plays a complex role in both physiological and pathological processes, influencing immune regulation, inflammation, and vascular remodeling. Structurally, it primarily exists as a homodimer, where each monomer contains a carbohydrate recognition domain (CRD) responsible for binding β-galactoside-containing glycoconjugates. This interaction enables Gal-1 to influence glycoprotein and glycolipid signaling in various cellular environments, including the extracellular matrix (ECM) [11]. Gal-1’s functional versatility depends on its cellular context and binding partners, impacting biological processes such as cell adhesion, migration, apoptosis, immune regulation, angiogenesis, and inflammation [12]. For example, its interaction with the leukocyte activation receptor CD69 influences T-cell differentiation, reinforcing its regulatory role in immune responses [13]. Furthermore, Gal-1 signaling in leukocytes depends on the expression of complex-type N-glycans, further highlighting its significance in immune cell function [14]. Its expression is tightly regulated by environmental signals and disease states, ensuring cellular homeostasis while contributing to disease pathogenesis [11]. Age and sex significantly influence Gal-1 expression and metabolism, contributing to variations across different tissues and disease susceptibility. Age-related shifts in Gal-1 levels have been widely documented in bone metabolism, immune function, and vascular health. Epigenetic modifications, such as DNA methylation in its promoter region, have been shown to suppress Gal-1 expression in the bone marrow stromal cells (BMSCs) of aged mice, indicating a potential link between aging-related epigenetic changes and impaired bone homeostasis [15]. Likewise, Gal-1 deletion has been associated with reduced osteogenic potential, further underscoring its role in skeletal health [16]. Additionally, the dynamic regulation of Gal-1 expression has been observed in immune system development, with levels rising in preterm infants due to amniotic infection and declining rapidly in the neonatal stages [17]. Moreover, alterations in Gal-1 expression in cochlear tissues of aging C57BL/6 mice suggest a potential link to age-related auditory decline [18]. In cardiovascular pathology, Gal-1’s involvement in age-related endothelial dysfunction and immune aging highlights its potential role in CVD progression. Understanding these mechanisms could inform therapeutic strategies aimed at mitigating vascular deterioration associated with aging. Sex-based disparities in Gal-1 levels have also been noted, particularly in CVDs. For instance, significant differences in its expression have been reported in human calcific aortic stenosis, where cardioprotective effects appear to vary between male and female patients [19]. Similarly, associations between serum Gal-1 levels and large artery atherosclerotic stroke suggest a potential influence of sex hormones on Gal-1 metabolism and disease susceptibility. Studies on osteoclastic activity further support its physiological significance, demonstrating that the reduced form of Gal-1 suppresses osteoclast differentiation in human peripheral blood mononuclear cells and murine RAW264 cells [20]. Collectively, these findings indicate that Gal-1 metabolism is intricately regulated by age and sex, impacting disease susceptibility and physiological adaptations across various life stages. Future research should explore hormonal regulation, epigenetic modifications, and their implications in Gal-1-mediated pathological processes. A deeper understanding of these variations could help inform personalized therapeutic strategies for age- and sex-related diseases involving Gal-1 dysregulation.

### 2.2. Dual Role of Galectin-1 in Cardiovascular Diseases

Gal-1 plays a complex role in CVDs, exerting both protective and pathological effects depending on its concentration and disease stage (Table 1). At lower levels, Gal-1 contributes to vascular homeostasis by modulating calcium channel activity, reducing arterial constriction, and alleviating hypertension [21,22]. Additionally, it supports endothelial integrity and immune regulation, reducing inflammation and aiding tissue repair. However, elevated Gal-1 levels are linked to adverse outcomes, including increased vascular inflammation and the progression of abdominal aortic aneurysm, highlighting its pro-inflammatory effects [23]. Moreover, higher serum concentrations correlate with a greater risk of mortality and cardiovascular complications [24,25]. The impact of Gal-1 in CVDs is highly context-dependent, influenced by both concentration and duration of exposure. While at physiological levels, Gal-1 limits immune activation and promotes endothelial repair, excessive expression triggers immune cell recruitment and amplifies inflammatory pathways, accelerating disease progression [26]. This delicate balance underscores the importance of targeted therapeutic approaches that optimize Gal-1’s effects without exacerbating pathology. Timing also plays a crucial role in determining its cardiovascular impact. During acute inflammatory responses, Gal-1 aids resolution by regulating leukocyte adhesion and apoptosis, limiting excessive immune activation and tissue damage [5]. In contrast, prolonged elevation in chronic conditions contributes to maladaptive cardiac remodeling, fibrosis, and extracellular matrix deposition, leading to ventricular stiffening and diastolic dysfunction, exacerbating heart failure progression [24]. Understanding Gal-1’s shifting role in cardiovascular pathology highlights the need for precise modulation strategies tailored to disease severity and progression. Future therapeutic approaches must account for its context-dependent effects to maximize benefits while minimizing potential risks.

Table 1 presents the dual roles of galectin-1 (Gal-1) in cardiovascular diseases, distinguishing its protective and pathological effects for clearer comparison. The protective functions of Gal-1 contribute to cardiovascular homeostasis by supporting endothelial integrity, reducing inflammation, and mitigating adverse remodeling processes. Conversely, its pathological effects drive disease progression, exacerbating vascular dysfunction, inflammation, fibrosis, and myocardial damage. The references cited provide supporting evidence for each function.

#### 2.2.1. Protective Effect

**Figure 1:**
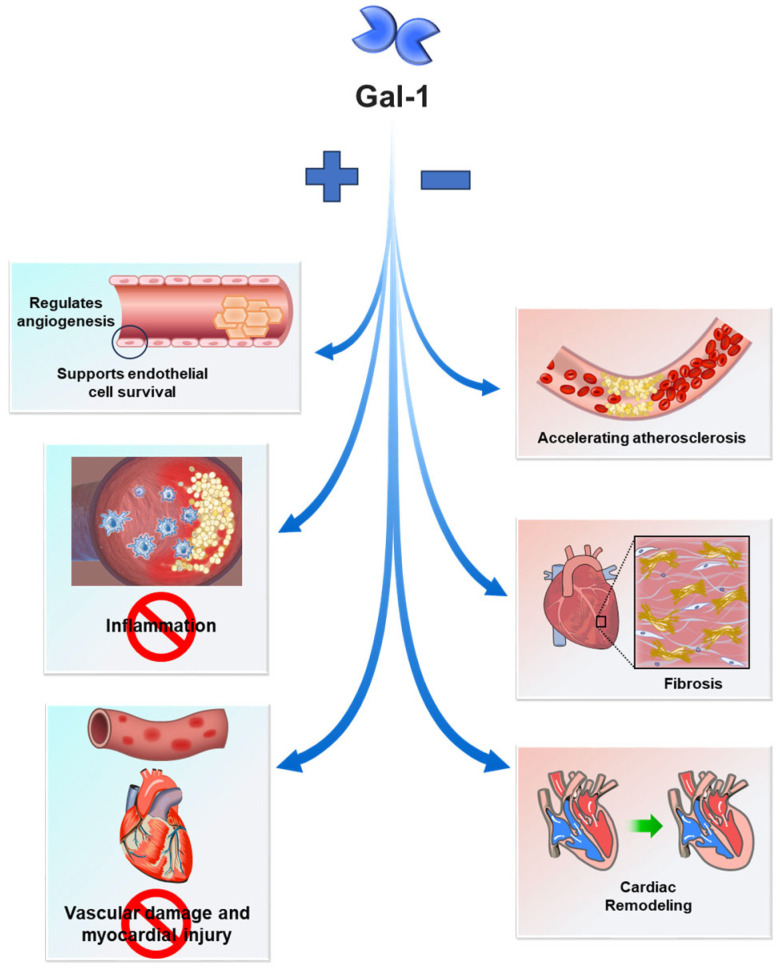
Dual role of galectin-1 in cardiovascular disease pathogenesis. Galectin-1 (Gal-1) plays a complex role in cardiovascular diseases (CVDs), exhibiting both protective and pathological effects depending on the context. On the beneficial side, Gal-1 supports endothelial cell survival, regulates angiogenesis, suppresses inflammation, and modulates immune responses to mitigate vascular damage and myocardial injury. Conversely, Gal-1 can also drive disease progression by fostering vascular inflammation, accelerating atherosclerosis, inducing fibrosis, and exacerbating adverse cardiac remodeling. This figure illustrates the nuanced, context-dependent functions of Gal-1 in CVD pathogenesis, underscoring its potential as both a biomarker for disease progression and a target for therapeutic intervention. In the diagram, “+” denotes beneficial effects, while “−” signifies pathological contributions.

**Figure 2:**
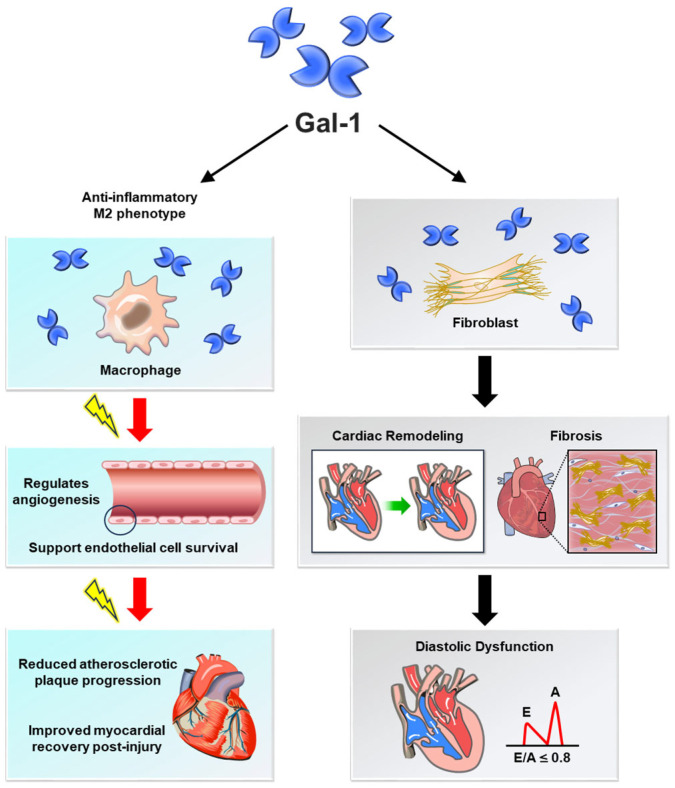
Molecular mechanisms of galectin-1 in cardiovascular diseases. Galectin-1 (Gal-1) exerts its effects in cardiovascular diseases (CVDs) through multiple molecular mechanisms. On the protective side, Gal-1 inhibits pro-inflammatory cytokine release, promotes macrophage polarization toward an anti-inflammatory M2 phenotype, and enhances endothelial cell survival and angiogenesis. These actions contribute to vascular repair, reduced atherosclerotic plaque progression, and improved myocardial recovery post-injury. However, Gal-1 can also facilitate pathological processes, such as enhancing fibroblast activation, increasing extracellular matrix deposition, and promoting cardiac fibrosis, which contribute to adverse ventricular remodeling and heart failure progression. Gal-1 influences calcium channel activity, affecting vascular tone and blood pressure regulation. This figure provides an overview of Gal-1’s molecular interactions with key cellular pathways in the cardiovascular system, emphasizing its complex and context-dependent roles.

Gal-1 plays a complex role in CVDs, exhibiting both protective and detrimental effects depending on disease stage and context (Figure 1). Its ability to regulate inflammation, vascular function, and tissue remodeling underscores its significance in cardiovascular pathology. One of its key protective mechanisms is the suppression of pro-inflammatory cytokines and adhesion molecules, which helps reduce vascular inflammation and slow atherosclerosis progression [19]. Moreover, Gal-1 supports endothelial cell survival and promotes angiogenesis, aiding in vascular repair and regeneration following injury [19]. It also plays a role in maintaining blood pressure by interacting with Ca(V)1.2, a protein essential for calcium channel activity and vascular tone regulation, thereby mitigating hypertensive damage [21,22]. Beyond its role in vascular function, Gal-1 exhibits anti-fibrotic properties, modulating fibrogenic cell activity and extracellular matrix remodeling (Figure 2). These actions help prevent adverse cardiac remodeling and fibrosis, both of which contribute to heart failure progression [19]. Furthermore, Gal-1 supports immune regulation by promoting a tolerogenic environment, reducing autoimmune-mediated cardiovascular damage [19]. Given these diverse functions, Gal-1 presents an opportunity for therapeutic targeting. Modulating its activity could help preserve vascular homeostasis, prevent atherosclerosis, and promote cardiac repair. However, its dual nature—where excessive expression can exacerbate inflammation and fibrosis—emphasizes the need for precision in therapeutic strategies. Future research should focus on optimizing Gal-1 modulation to maximize benefits while minimizing potential risks.

#### 2.2.2. Pathological Implications

Gal-1 not only exerts protective effects in CVDs, but can also contribute to pathological processes, particularly in chronic inflammation, vascular remodeling, and fibrosis. While its anti-inflammatory actions are well documented, under certain conditions, Gal-1 paradoxically promotes disease progression. In chronic inflammation, Gal-1 may enhance immune cell recruitment and activation, intensifying inflammatory responses within the vasculature and myocardium [28,40]. Moreover, it has been implicated in immune cell polarization, with studies suggesting that it can drive a pro-inflammatory M1 phenotype over an anti-inflammatory M2 phenotype, exacerbating tissue injury [28,29]. Beyond inflammation, Gal-1 plays a significant role in fibrosis and adverse cardiac remodeling by influencing fibroblast activity and extracellular matrix deposition, contributing to maladaptive structural changes in the myocardium [23,24]. Elevated Gal-1 levels have been associated with increased mortality risk and adverse cardiovascular outcomes, making it a potential biomarker for disease progression. Several cohort studies have examined its prognostic value, identifying quantitative thresholds, hazard ratios (HRs), and confidence intervals (CIs) that support its role in cardiovascular risk stratification (Table 2). For instance, critically ill patients with serum Gal-1 levels in the highest tertile (≥71 ng/mL) exhibited significantly increased 90-day mortality compared to those in the lowest tertile (<39 ng/mL), with an adjusted HR of 3.21 (95% CI: 1.90–5.42, *p* < 0.001) [25]. Similarly, elevated Gal-1 levels were linked to a higher incidence of acute kidney injury (AKI) within 48 h of intensive care unit admission (adjusted HR: 2.88, 95% CI: 1.20–6.88, *p* = 0.017) [25]. In coronary artery disease (CAD), higher circulating Gal-1 levels correlated with increased fibrosis markers and vascular stiffening, with a median serum concentration of 56.3 ng/mL in patients with severe CAD compared to 32.1 ng/mL in controls (*p* < 0.01) [24]. Furthermore, in heart failure (HF) cohorts, elevated Gal-1 levels were associated with worsening left ventricular diastolic dysfunction and increased hospitalization rates, with mean levels of 68.5 ng/mL in HF patients versus 40.2 ng/mL in non-HF individuals (HR: 2.45, 95% CI: 1.62–3.71, *p* < 0.001) [37]. Table 2 provides an overview of key human cohort studies investigating Gal-1 as a biomarker in CVDs, organized by disease entity, sample size, assay type, median/mean Gal-1 levels, and clinical outcomes. Physiological levels of Gal-1 (typically 20–50 ng/mL) are linked to its beneficial effects, including immune modulation, endothelial protection, and anti-inflammatory signaling [41]. At these concentrations, Gal-1 supports angiogenesis, enhances vascular integrity, and suppresses excessive immune activation, contributing to cardiovascular homeostasis [6]. However, when plasma levels exceed 70–100 ng/mL, its role shifts toward pro-inflammatory and pro-fibrotic pathways, exacerbating disease progression [25]. Elevated concentrations have been associated with increased vascular permeability, leukocyte recruitment, and fibrosis, particularly in conditions such as atherosclerosis, myocardial infarction, and heart failure [24]. In critically ill patients, serum Gal-1 levels above 90 ng/mL were linked to a threefold increase in mortality risk (HR: 3.21, 95% CI: 1.90–5.42, *p* < 0.001) [37]. The mechanistic basis for this concentration-dependent shift lies in Gal-1’s interactions with glycan-binding partners and immune receptors. At lower concentrations, Gal-1 preferentially binds to anti-inflammatory glycoproteins, fostering immune tolerance and tissue repair [42]. Conversely, higher concentrations activate pro-inflammatory signaling pathways, driving fibroblast activation, extracellular matrix deposition, and maladaptive cardiac remodeling [43]. Further research is needed to define precise clinical cutoffs for Gal-1 levels in various cardiovascular conditions and to develop therapeutic strategies for modulating its expression. These findings emphasize the complex and context-dependent nature of Gal-1 in CVDs, underscoring its potential as both a biomarker and a therapeutic target.

Recent studies provide further insights into Gal-1 and its role in vascular pathology, particularly its involvement in both atherogenesis and fibrosis. Evidence from an abdominal aortic aneurysm model demonstrated that elevated Gal-1 levels in angiotensin II-infused apolipoprotein E-deficient mice correlated with increased matrix metalloprotease-9 activity and extracellular matrix degradation [23]. Gal-1 deletion in this model led to reduced aneurysm severity and inflammation, suggesting that excessive expression may compromise vascular integrity by amplifying tumor necrosis factor-alpha (TNF-α)-driven inflammatory responses in vascular smooth muscle cells and adventitial fibroblasts [23]. Conversely, data from CAD cohorts indicate a strong correlation between elevated circulating Gal-1 levels and markers of fibrosis, vascular stiffening, and maladaptive remodeling [25]. Mechanistically, Gal-1 interacts with transforming growth factor-beta signaling, contributing to fibroblast activation and excessive collagen deposition, which in turn impairs vascular compliance and exacerbates ischemic injury [29]. While Gal-1 may support tissue repair under acute conditions, sustained elevation appears to drive pathological fibrosis in chronic cardiovascular diseases. The conflicting roles of Gal-1 highlight key factors influencing its effects, including dose dependence, post-translational modifications, and glycan interactions. At lower concentrations, Gal-1 promotes endothelial integrity and immune regulation, whereas excessive expression fosters inflammatory signaling and fibrosis [23,39]. Additionally, post-translational modifications—such as oxidation—can alter Gal-1’s functional properties, affecting its interactions with immune cells and extracellular matrix components [44]. Variations in glycan expression across vascular compartments further shape its binding affinity, influencing disease progression [11]. Given these context-dependent effects, therapeutic strategies must be carefully tailored to optimize benefits while mitigating risks. Targeting Gal-1 for aneurysm prevention may require the selective inhibition of its inflammatory and matrix-degrading properties, whereas interventions for CAD and heart failure should focus on modulating its fibrotic activity. Future research should explore precision medicine approaches that account for disease-specific Gal-1 dynamics, enabling its therapeutic potential while minimizing adverse outcomes.

Table 2 presents key human cohort studies investigating the role of galectin-1 (Gal-1) as a biomarker in cardiovascular diseases. It stratifies available studies by disease entity, sample size, assay type used to quantify Gal-1 levels, median or mean Gal-1 concentrations observed in patient cohorts, and associated clinical endpoints. Moreover, hazard ratios (HRs) with confidence intervals (CIs) are included where available to highlight the strength of associations between elevated Gal-1 levels and adverse cardiovascular outcomes.

## 3. Insights from Recent Studies

### 3.1. Galectin-1 and Atherosclerosis

**Figure 3:**
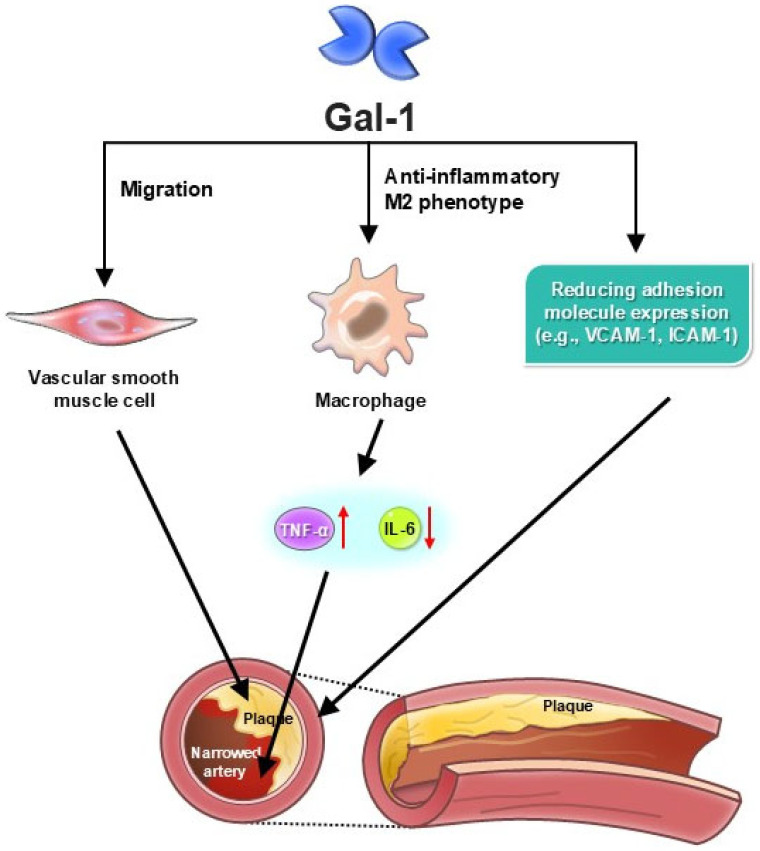
Galectin-1 in atherosclerosis progression and plaque stability. Galectin-1 (Gal-1) influences multiple stages of atherosclerosis, exhibiting both protective and pathological effects. In early atherosclerosis, Gal-1 modulates endothelial function by reducing adhesion molecule expression (e.g., VCAM-1, ICAM-1), limiting leukocyte adhesion and infiltration into the vascular wall. It also promotes macrophage polarization toward an anti-inflammatory M2 phenotype, reducing pro-inflammatory cytokines such as IL-6 and TNF-α. These actions contribute to plaque stabilization and reduced atherosclerotic burden. However, under certain conditions, Gal-1 may enhance foam cell formation, promote vascular smooth muscle cell migration, and contribute to plaque progression. Additionally, Gal-1 binds to lipoprotein(a), which may influence lipid metabolism and atherogenesis. This figure illustrates the multifaceted role of Gal-1 in atherosclerosis development, highlighting its potential as a therapeutic target for stabilizing vulnerable plaques and reducing cardiovascular risk.

Recent studies have provided deeper insights into Gal-1’s role in atherosclerosis, demonstrating its involvement in several key pathological processes (Figure 3). These include endothelial dysfunction, macrophage activation, foam cell formation, and vascular inflammation, all of which contribute to disease progression. Gal-1 influences endothelial cell function by regulating adhesion molecule expression, leukocyte adhesion, and barrier integrity, affecting early atherosclerotic changes [27]. Moreover, it plays a crucial role in immune modulation by directing macrophage polarization toward an anti-inflammatory M2 phenotype, which may reduce plaque inflammation and enhance plaque stability [29]. Beyond its immunoregulatory functions, Gal-1 binds to lipoprotein(a), a key factor in atherogenesis, further implicating it in disease pathophysiology [35]. Understanding these interconnected roles of Gal-1 in atherosclerosis underscores the importance of targeted therapeutic approaches that balance its protective effects while mitigating its contribution to disease progression. Further research into its regulatory mechanisms could provide new avenues for intervention, particularly in terms of modulating immune responses and improving vascular integrity.

#### 3.1.1. Anti-Inflammatory Actions

Recent research highlights Gal-1’s role in mitigating inflammation in atherosclerosis, demonstrating its ability to regulate immune responses within plaques. By suppressing key pro-inflammatory cytokines, including interleukin-6 (IL-6), TNF-α, and interleukin-1 beta (IL-1β), Gal-1 reduces inflammatory signaling in macrophages and endothelial cells, limiting immune activation in the vascular environment [28]. In addition to cytokine suppression, Gal-1 inhibits the adhesion and migration of monocytes and lymphocytes to the vascular endothelium, preventing excessive immune cell recruitment into plaques [33]. This restriction helps maintain endothelial integrity and reduces plaque vulnerability. Furthermore, Gal-1 promotes macrophage polarization toward the anti-inflammatory M2 phenotype, increasing interleukin-10 (IL-10) expression while downregulating pro-inflammatory mediators [13]. These combined actions contribute to chronic inflammation resolution, enhancing plaque stability and slowing disease progression. By regulating immune activity and promoting a protective cellular environment, Gal-1 emerges as a potential target for therapeutic strategies aimed at reducing atherosclerotic risk and improving cardiovascular outcomes.

#### 3.1.2. Endothelial Cell Function Modulation

Recent studies have provided deeper insights into Gal-1’s role in endothelial cell function, particularly in the context of atherosclerosis. By regulating adhesion molecules such as vascular cell adhesion molecule-1 (VCAM-1) and intercellular adhesion molecule-1 (ICAM-1), Gal-1 influences leukocyte adhesion and trans-endothelial migration, processes essential for atherosclerotic plaque development [45]. Beyond its role in immune cell recruitment, Gal-1 affects endothelial cell proliferation and apoptosis, impacting barrier function and vascular permeability [29]. It also supports endothelial cell survival and angiogenesis, contributing to vascular integrity and repair following injury [34]. These combined functions illustrate Gal-1’s significance in both maintaining endothelial homeostasis and influencing atherosclerosis progression. Understanding these regulatory mechanisms provides further evidence of Gal-1’s dual role in vascular health, reinforcing its potential as both a therapeutic target and a key factor in disease pathogenesis.

### 3.2. Galectin-1 and Myocardial Infarction

**Figure 4:**
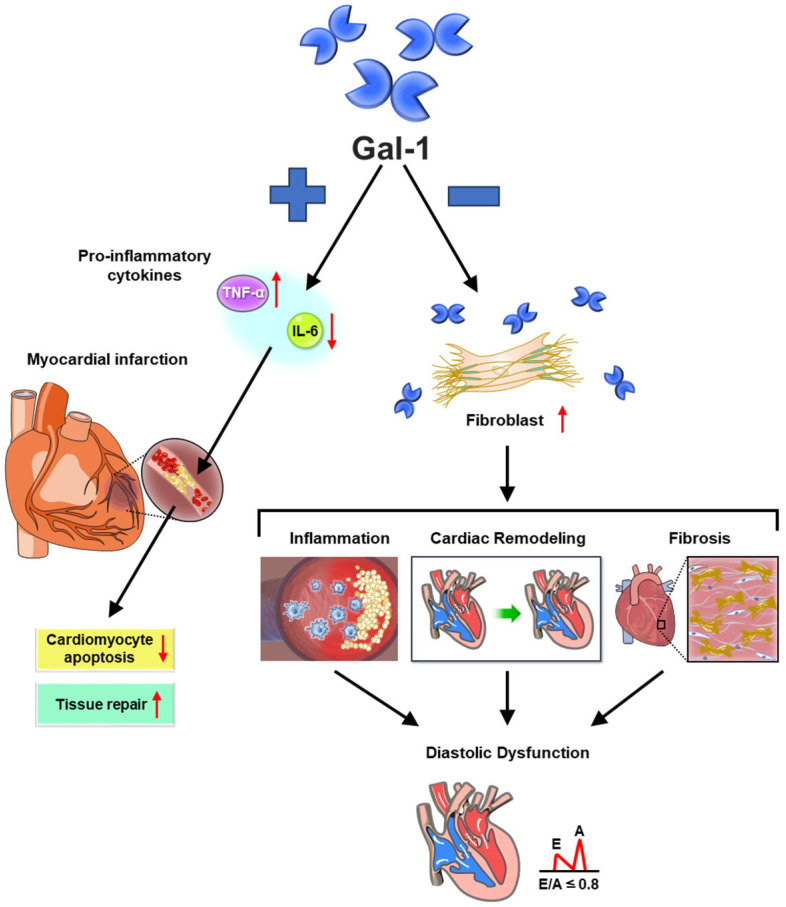
Galectin-1 in myocardial infarction and heart failure progression. Galectin-1 (Gal-1) plays a dual role in myocardial infarction (MI) and heart failure (HF) by regulating inflammatory responses, fibrosis, and tissue remodeling. After MI, Gal-1 exerts cardioprotective effects by suppressing pro-inflammatory cytokines (e.g., TNF-α, IL-6), reducing cardiomyocyte apoptosis, and promoting angiogenesis to facilitate tissue repair. However, in chronic heart failure, Gal-1 can contribute to pathological cardiac remodeling by stimulating fibroblast activation, increasing extracellular matrix deposition, and promoting myocardial fibrosis, which leads to ventricular stiffening and diastolic dysfunction. Additionally, Gal-1 influences immune cell polarization, affecting macrophage-mediated inflammation and cardiac healing. This figure illustrates the complex and context-dependent actions of Gal-1 in MI and HF, emphasizing its potential as a biomarker and therapeutic target in cardiovascular diseases. In the diagram, “+” denotes beneficial effects, while “−” signifies pathological contributions. The arrow-up indicates an increase, while the arrow-down represents a decrease.

Gal-1 plays a multifaceted role in myocardial infarction (MI), contributing to both acute injury response and long-term cardiac remodeling (Figure 4). Studies indicate that Gal-1 mitigates ischemia–reperfusion injury by reducing inflammation and preventing cardiomyocyte apoptosis, underscoring its protective effects during the early phase of MI [30]. Beyond acute injury, Gal-1 influences post-MI cardiac inflammation and ventricular remodeling, highlighting its potential as a therapeutic target for managing long-term cardiac pathology [31]. Moreover, it plays a role in cardiac hypertrophy by modulating the splice-variant-specific activity of the Ca(V)1.2 calcium channel, which may impact structural adaptations following MI [32]. Together, these findings illustrate the complex and context-dependent nature of Gal-1 in MI, reinforcing the need for targeted therapeutic strategies that optimize its benefits while minimizing potential pathological effects.

#### 3.2.1. Cardioprotective Mechanisms

Recent studies have highlighted Gal-1’s cardioprotective role in MI, demonstrating its ability to mitigate ischemic injury and preserve cardiac function. Its anti-inflammatory properties help suppress pro-inflammatory cytokines, facilitating inflammation resolution within the infarcted myocardium [31]. Moreover, Gal-1 reduces oxidative stress and prevents cardiomyocyte apoptosis, contributing to tissue integrity and post-MI recovery [30]. Beyond its role in inflammation control, Gal-1 promotes angiogenesis, supporting neovascularization—a crucial process for tissue repair following MI [44]. These combined effects position Gal-1 as a promising therapeutic target for reducing myocardial damage and improving clinical outcomes in patients with coronary artery disease. Given its ability to regulate multiple protective pathways, optimizing Gal-1 modulation may offer new intervention strategies for MI management.

#### 3.2.2. Fibrosis and Remodeling

Fibrosis plays a crucial yet complex role in MI recovery. While excessive fibrosis can lead to adverse cardiac remodeling and impaired function, a controlled fibrotic response is essential for structural integrity, preventing chamber rupture and facilitating tissue repair [46]. Gal-1 serves as a key regulator of fibrosis and ECM remodeling following MI. It modulates fibroblast activation, influencing collagen deposition and ECM turnover to stabilize infarcted myocardia [47]. Studies suggest that early post-MI Gal-1 expression supports scar formation, reducing ventricular rupture risk while preserving cardiac architecture [48]. However, prolonged overexpression may lead to excessive fibroblast proliferation, resulting in ECM accumulation, ventricular stiffening, and heart failure progression [26]. Beyond its direct effects on fibroblasts, Gal-1 interacts with matrix metalloproteinases (MMPs) and tissue inhibitors of metalloproteinases (TIMPs), maintaining a balance between ECM degradation and synthesis to prevent maladaptive remodeling [49]. This regulatory function ensures that fibrosis remains adaptive rather than excessive, supporting myocardial repair while mitigating long-term dysfunction. Given its context-dependent effects, Gal-1 represents a promising therapeutic target for optimizing post-MI fibrosis. Strategies that fine-tune its activity could enhance beneficial scar formation while limiting pathological ECM accumulation, opening new possibilities for precision medicine in ischemic heart disease management.

### 3.3. Galectin-1 and Heart Failure

Recent studies have highlighted Gal-1’s role in heart failure (HF), particularly its association with left ventricular diastolic dysfunction and heart failure with preserved ejection fraction (HFpEF) [37]. Evidence indicates that Gal-1 contributes to cardiac fibrosis and remodeling, reinforcing its involvement in HF pathophysiology [36]. Additionally, Gal-1’s ability to modulate immune responses may further influence HF progression [40,50]. These findings underscore Gal-1’s significance in HF development and its potential as a therapeutic target for managing HFpEF and associated diastolic dysfunction (Figure 4). Future research should focus on refining strategies to harness its regulatory mechanisms, offering new avenues for therapeutic intervention.

#### 3.3.1. Role in Cardiac Fibrosis

Gal-1 plays a complex role in cardiac fibrosis, influencing both its initiation and progression through interactions with immune cells, fibroblasts, and ECM components. Following myocardial injury, Gal-1 modulates inflammatory responses, facilitating monocyte and macrophage recruitment, which in turn secrete pro-fibrotic cytokines such as transforming growth factor-beta (TGF-β) [26]. This cytokine is a key driver of fibroblast activation, promoting their differentiation into myofibroblasts responsible for collagen synthesis and ECM deposition [51]. As fibrosis advances, Gal-1 interacts with fibroblasts and endothelial cells, regulating collagen production and ECM remodeling. Specifically, it enhances TGF-β/Smad signaling, amplifying fibrotic responses by increasing fibroblast proliferation and collagen deposition [52]. Moreover, Gal-1 modulates MMPs and tissue inhibitors of metalloproteinases (TIMPs), balancing ECM degradation and synthesis to prevent maladaptive remodeling [53]. While the early activation of MMPs supports scar formation and infarct stabilization, sustained Gal-1 expression can lead to excessive ECM accumulation, contributing to ventricular stiffening and heart failure progression. Interestingly, Gal-1 also exhibits anti-fibrotic properties under certain conditions, facilitating ECM degradation and fibrosis resolution through proteolytic enzyme activation [54]. This duality underscores the need for the precise therapeutic modulation of Gal-1 to balance scar formation with tissue remodeling, ultimately preventing adverse cardiac outcomes.

#### 3.3.2. Impact on Immune Response

Recent research underscores Gal-1’s critical role in shaping immune responses in HF [40]. By modulating immune cell activity within the myocardium, Gal-1 contributes to an immunosuppressive microenvironment that may influence disease progression [55]. Gal-1 regulates T-cell dynamics by promoting apoptosis in effector T cells while enhancing regulatory T-cell (Treg) survival, reinforcing immune tolerance and limiting excessive inflammatory damage [55]. Moreover, it influences immune homeostasis by directing macrophage polarization toward an anti-inflammatory M2 phenotype, a process associated with tissue repair [56]. Dysregulated immune responses contribute to myocardial inflammation and adverse cardiac remodeling, making Gal-1’s immunomodulatory effects particularly relevant in HF pathology [26]. Beyond direct immune cell regulation, Gal-1 suppresses the activation and proliferation of effector T cells while promoting Treg expansion, reducing excessive immune responses and minimizing myocardial injury [55]. It also regulates the secretion of pro-inflammatory cytokines and chemokines, such as TNF-α and IL-6, thereby mitigating inflammation and tissue damage in the failing heart [26]. These findings highlight the intricate relationship between Gal-1 and immune regulation in HF, reinforcing its potential as a therapeutic target. Modulating Gal-1 activity could provide a novel approach to reducing inflammation and preserving cardiac function in patients with HF.

## 4. Clinical Implications and Therapeutic Potential

### 4.1. Galectin-1 as a Biomarker for Cardiovascular Risk Assessment

Gal-1 has gained recognition as a potential biomarker for cardiovascular risk assessment, with clinical evidence linking its levels to key risk factors such as hypertension, dyslipidemia, and insulin resistance. Elevated Gal-1 concentrations correlate with the severity of conditions like atherosclerosis, myocardial infarction, and heart failure, reinforcing its prognostic relevance [50]. Beyond traditional risk indicators, Gal-1 assessment offers valuable insights into identifying individuals at higher risk of cardiovascular events [25]. Additionally, its measurement may aid in monitoring disease progression and evaluating treatment responses in cardiovascular patients [38]. The development of highly sensitive assays for detecting Gal-1 enhances its feasibility for clinical integration, enabling refined risk stratification and personalized therapeutic strategies. Incorporating Gal-1 into routine cardiovascular assessments could improve early intervention efforts and help mitigate disease burden. However, further research is needed to establish its clinical utility, validate optimal cutoff levels, and clarify its mechanistic role in cardiovascular pathophysiology, potentially shaping future therapeutic approaches.

### 4.2. Targeting Galectin-1 for Therapeutic Intervention

Targeting Gal-1 as a therapeutic strategy presents promising opportunities for managing cardiovascular diseases. Preclinical studies indicate that inhibiting Gal-1 can slow atherosclerosis progression, reduce myocardial infarction-induced injury, and prevent adverse cardiac remodeling by modulating inflammation, endothelial dysfunction, and fibrosis [57,58]. Pharmacological approaches, including Gal-binding ligands and non-carbohydrate inhibitors, have shown potential in reducing cardiovascular morbidity and mortality [59,60]. Additionally, the development of specific inhibitors and monoclonal antibodies against Gal-1 offers exciting prospects for precision medicine, enabling tailored treatments for individual patients [61,62]. Despite these advancements, further research is required to refine therapeutic strategies, establish optimal dosage regimens, and evaluate long-term safety profiles in clinical settings [15,63]. Addressing these gaps will be essential for translating Gal-1-targeted interventions into viable treatments for cardiovascular disease management.

#### 4.2.1. Pharmacological Agents

The pharmacological inhibition of Gal-1 presents a promising strategy for treating cardiovascular diseases, with several small molecules and monoclonal antibodies demonstrating efficacy in preclinical models of atherosclerosis, myocardial infarction, and heart failure [58,60]. These agents work by disrupting Gal-1-mediated interactions, reducing pro-inflammatory signaling, alleviating endothelial dysfunction, and minimizing adverse cardiac remodeling [61,63]. Despite encouraging preclinical findings, significant challenges remain in translating Gal-1 inhibitors into viable clinical therapies. One of the most studied inhibitors, OTX008, has shown potential in targeting Gal-1-driven tumor progression and inflammation. However, its pharmacokinetic limitations—including rapid plasma clearance and poor bioavailability—may hinder its systemic therapeutic efficacy [62]. Additionally, its short elimination half-life suggests that frequent dosing may be necessary, raising concerns about patient compliance and long-term safety [59,64,65]. Improving molecular designs to enhance selectivity while minimizing off-target effects remains a crucial focus of therapeutic development. Furthermore, comprehensive data on target engagement for Gal-1 inhibitors is lacking. While preclinical studies have shown reductions in Gal-1 activity, quantitative assessments of full target occupancy and downstream signaling effects remain limited [66]. Without robust pharmacodynamic data, determining optimal dosing strategies, therapeutic windows, and long-term efficacy in cardiovascular applications remains challenging. Future research should prioritize biomarker development to track Gal-1 inhibition in vivo, refining dosing regimens to maximize therapeutic benefit while minimizing adverse effects. Advancing Gal-1-targeted pharmacotherapy into clinical use will require the optimization of drug pharmacokinetics, improved specificity, and reliable measures of target engagement to ensure efficacy and safety.

#### 4.2.2. Gene Therapy Approaches

Gene therapy offers a promising approach for regulating Gal-1 expression in cardiovascular diseases, enabling precise and sustained modulation within affected tissues. Utilizing viral vectors such as adeno-associated viruses (AAVs) and lentiviruses, as well as non-viral delivery systems like lipid nanoparticles and electroporation, gene therapy can facilitate either the upregulation or suppression of Gal-1, depending on therapeutic objectives [15,63]. Strategies to enhance Gal-1 expression through engineered gene constructs have been explored for their protective effects, including immune modulation, angiogenesis promotion, and reducing ischemia–reperfusion injury [30,31]. Conversely, RNA interference (RNAi) and CRISPR-Cas9-mediated gene editing have been investigated to suppress Gal-1 in pathological conditions where excessive activity contributes to fibrosis, vascular remodeling, or chronic inflammation [5,26,37]. Preclinical studies suggest that fine-tuning Gal-1 levels using these approaches may mitigate disease progression in atherosclerosis, myocardial infarction, and heart failure [5,19,27]. Despite its therapeutic potential, gene therapy faces significant delivery challenges, particularly regarding AAV vector tropism and immunogenicity. While AAV vectors achieve long-term gene expression with minimal genomic integration risk, conventional serotypes (e.g., AAV1, AAV9) exhibit suboptimal tropism for cardiomyocytes, resulting in inefficient transduction and off-target accumulation in non-cardiac tissues such as the liver [67]. Engineered cardiotropic AAV variants like AAVrh.74 and C102 have demonstrated improved cardiac gene delivery while reducing immunogenicity in non-human primates [43]. Additionally, nanoparticle-based enhancers have been explored to optimize cardiac tropism and minimize systemic distribution, enhancing therapeutic efficacy [68]. Immune responses to AAV vectors present another challenge, potentially limiting transduction efficiency and long-term gene expression. Pre-existing neutralizing antibodies against AAV capsids can significantly reduce vector uptake, necessitating strategies such as immune evasion techniques, capsid engineering, and transient immunosuppression to improve delivery [65]. Furthermore, repeated AAV administration may trigger adaptive immune responses, requiring careful dosing strategies and alternative delivery approaches to sustain therapeutic effects. To ensure cardiovascular-specific gene expression and minimize off-target effects, promoter selection is critical. The cardiac troponin T promoter has been widely used for cardiomyocyte-specific expression, ensuring targeted gene delivery within the heart [64]. Other regulatory elements, such as the myosin heavy chain promoter and natriuretic peptide precursor A enhancer, have also been employed to restrict transgene expression to cardiac tissues while avoiding unintended activation in non-cardiac cells [66]. These promoters provide a robust framework for optimizing gene therapy strategies tailored to cardiovascular applications. Gene therapy offers long-lasting and localized therapeutic benefits, potentially reducing the need for repeated drug administration. However, significant challenges remain, including immune responses to viral vectors, off-target genetic modifications, and regulatory hurdles that must be addressed to ensure safety and efficacy in clinical applications [15,63]. Further research is needed to refine delivery mechanisms, enhance tissue-specific targeting, and evaluate long-term outcomes in preclinical and clinical settings. Advancing these efforts will be essential to determine the feasibility of translating Gal-1-directed gene therapy into viable cardiovascular treatments.

## 5. Challenges and Future Directions

### 5.1. Limitations of Current Research

Despite significant progress in understanding Gal-1 role in CVDs, several challenges remain in fully characterizing its function and translating findings into clinical applications. One major issue is the reliance on animal models and in vitro studies, which, while valuable, may not fully capture the complexity of human cardiovascular pathology [1]. Species-specific differences in immune regulation, metabolism, and cardiovascular remodeling raise concerns regarding the direct translation of findings to human patients [5,27]. Additionally, inconsistencies in experimental conditions—such as variations in disease models and Gal-1 expression levels—complicate efforts to draw broad conclusions applicable to diverse patient populations. Another limitation is the tendency of current research to focus on isolated aspects of cardiovascular pathology—such as atherosclerosis, myocardial infarction, or heart failure—without fully considering the interplay between these conditions [24,37]. Given that CVDs often involve interconnected pathological processes, a more integrated approach is needed to clarify how Gal-1 contributes to disease progression across cardiovascular compartments and patient subgroups. This is especially important in the context of metabolic risk factors, which significantly influence CVD development, as highlighted by the Global Burden of Disease Study [8]. Investigating Gal-1’s interactions with metabolic dysregulation, inflammation, and vascular remodeling remains a priority for future research. The safety and efficacy of targeting Gal-1 in clinical settings also remain uncertain. While preclinical studies suggest potential therapeutic benefits—including reducing inflammation, preventing vascular remodeling, and mitigating myocardial injury [26,30]—translating these findings into viable human therapies presents challenges. Gal-1’s diverse biological functions, including immunomodulation, angiogenesis regulation, and ECM remodeling, could lead to unintended consequences when therapeutically manipulated [6]. Concerns regarding off-target effects and long-term risks underscore the need for rigorous safety evaluations before considering Gal-1-targeted therapies as viable treatment options. To overcome these limitations, future research should prioritize developing more human-relevant disease models, such as patient-derived organoids and advanced bioengineered systems, to better replicate the complexities of human CVDs [19]. Multi-omics approaches integrating genomics, proteomics, and metabolomics could provide a more comprehensive understanding of Gal-1’s role in cardiovascular pathology [10]. Additionally, large-scale clinical studies investigating circulating Gal-1 levels as potential biomarkers for disease progression and treatment response would clarify its clinical relevance [25,66]. Ultimately, well-designed clinical trials evaluating Gal-1-targeting agents—such as small-molecule inhibitors and biologics—are necessary to determine their safety, efficacy, and suitability for specific patient populations [64,65]. By addressing these challenges through interdisciplinary collaboration and innovative methodologies, future research can pave the way for Gal-1-based strategies that enhance cardiovascular medicine while minimizing potential risks.

### 5.2. Unresolved Questions and Areas for Further Investigation

Although significant progress has been made in understanding Gal-1’s role in CVDs, several key questions remain unresolved. One major challenge is pinpointing the precise molecular mechanisms by which Gal-1 influences cardiovascular pathogenesis. While research has established its involvement in inflammation, fibrosis, and endothelial function, the specific downstream signaling pathways and molecular interactions require further clarification [1,6]. Investigating how Gal-1 modulates intracellular signaling across various cardiovascular cell types—including endothelial cells, vascular smooth muscle cells, and immune cells—could provide deeper insights into its function [5,14]. Another unresolved issue is the context-dependent nature of Gal-1’s effects in cardiovascular health and disease. Some studies highlight its cardioprotective properties, particularly in immune modulation and inflammation suppression [26,28], whereas others indicate its contribution to pathological processes such as vascular remodeling and atherosclerosis [23,27]. Determining the conditions under which Gal-1 exerts protective versus harmful effects will be essential for evaluating its therapeutic potential. Additionally, the relationship between Gal-1 and metabolic risk factors in CVDs remains poorly defined. While diabetes and obesity are well-established contributors to cardiovascular disease [7,8], Gal-1’s precise role in metabolic dysregulation and its potential as a biomarker for cardiovascular risk require further investigation. Emerging evidence suggests that Gal-1 may influence lipid metabolism and vascular inflammation, but its mechanisms need to be elucidated [10,24]. The potential role of Gal-1 in platelet function and coagulation also presents an intriguing research avenue. Recent findings indicate an interaction with platelet factor 4 (CXCL4), raising questions about its involvement in platelet activation and thrombotic complications associated with CVDs [12]. A clearer understanding of these interactions could support the development of antithrombotic therapies targeting Gal-1-related pathways. Large-scale clinical studies are necessary to determine the prognostic and diagnostic value of circulating Gal-1 levels in cardiovascular patients. While elevated Gal-1 levels have been linked to increased risks of heart failure, kidney dysfunction, and coronary artery disease [24,37], these associations require validation across diverse populations. Parallel efforts to develop selective Gal-1 inhibitors, including antibody-based therapies and carbohydrate-binding domain inhibitors, are gaining interest [58,64]. However, rigorous testing is essential to assess their safety and efficacy before clinical implementation. Addressing these open questions will enhance our understanding of Gal-1’s complex role in CVDs and inform the development of targeted therapies that leverage its unique biological functions. Future research integrating multi-omics approaches, patient-derived models, and large-scale clinical trials will be instrumental in translating these findings into clinical applications.

Recent technological advancements have provided powerful tools for investigating Gal-1 in CVDs, offering new opportunities for precision medicine, biomarker discovery, and therapeutic development. Three key innovations—omics-based approaches, CRISPR screening, and bioengineering platforms—have deepened our understanding of Gal-1’s complex role in CVD pathogenesis. Omics technologies have revolutionized biomedical research by enabling high-resolution profiling of the molecular networks involved in disease progression. Spatial transcriptomics, which integrates transcriptomic analysis with tissue architecture mapping, allows researchers to visualize Gal-1 expression across different cardiovascular compartments with unprecedented detail [66]. Unlike bulk RNA sequencing, which averages gene expression across heterogeneous cell populations, spatial transcriptomics preserves tissue context, revealing microenvironment-specific regulatory patterns [41]. Combining spatial transcriptomics with single-cell RNA sequencing further refines this approach, distinguishing cell-type-specific roles of Gal-1 in atherosclerosis and heart failure [37]. CRISPR-based screening techniques provide a powerful toolset for functional genomics, enabling the systematic interrogation of Gal-1-associated pathways. CRISPR-Cas9 knockout and activation screens allow the targeted disruption or enhancement of Gal-1 expression, clarifying its direct involvement in inflammatory responses, endothelial function, and fibrosis [15]. CRISPR interference and CRISPR activation expand these capabilities, fine-tuning gene expression without permanent genomic alterations, making them particularly useful for studying the dose-dependent effects of Gal-1 modulation [65]. Additionally, CRISPR-generated disease models, including patient-derived induced pluripotent stem cells carrying Gal-1 mutations, enhance translational relevance by mimicking cardiovascular pathology [43]. Bioengineering platforms have also provided novel opportunities for modeling cardiovascular diseases and testing Gal-1-targeted interventions. Organ-on-chip systems, which replicate human tissue microenvironments, offer advanced alternatives to traditional animal models [67]. Vascular-on-chip models, integrating endothelial cells, pericytes, and immune components, simulate Gal-1-driven pathological mechanisms under physiologically relevant conditions, allowing the real-time monitoring of its effects on vascular integrity, inflammatory signaling, and tissue remodeling [60]. Moreover, bioprinting technologies have enabled the fabrication of 3D cardiac and vascular tissues embedded with patient-derived cells, offering a more accurate representation of disease progression and therapeutic response [6]. Integrating omics, CRISPR screening, and bioengineering tools will significantly enhance Gal-1 research, providing mechanistic insights that refine therapeutic strategies. Future studies leveraging these technologies will contribute to a more comprehensive understanding of Gal-1’s role in CVDs, ultimately advancing precision medicine for disease prevention and treatment.

### 5.3. Potential Strategies to Overcome Challenges

Advancing research on Gal-1 in CVDs requires a comprehensive approach integrating scientific advancements, preclinical and clinical studies, and global collaboration. Several strategies can accelerate progress in understanding Gal-1’s therapeutic potential and overcoming existing challenges. One key strategy involves leveraging advanced omics technologies, such as single-cell RNA sequencing and proteomics, to elucidate the molecular mechanisms underlying Gal-1’s role in cardiovascular pathology. Mapping Gal-1 interactions at the cellular and molecular levels can clarify its effects on immune modulation, vascular remodeling, and metabolic regulation [1,6]. Integrating these approaches with systems biology techniques, including network-based analyses and AI-driven modeling, may provide broader insights into Gal-1’s impact across different CVD subtypes. Developing physiologically relevant preclinical models is another crucial step. Traditional animal models often fail to fully replicate human cardiovascular diseases, limiting their translational potential. Advanced models, such as humanized mouse models, organ-on-chip systems, and induced pluripotent stem cell-derived cardiomyocytes, offer more reliable experimental platforms for studying Gal-1’s function and therapeutic applications [5,27]. Enhancing gene-editing technologies, such as CRISPR-Cas9, can further refine investigations into Gal-1’s regulatory mechanisms and therapeutic potential. International collaboration and data sharing are vital for advancing research and maximizing impact. Since Gal-1 is relevant to immunology, oncology, and cardiovascular research, fostering interdisciplinary partnerships will facilitate knowledge exchange and resource sharing. Large-scale initiatives, such as the Global Burden of Disease Study, provide valuable epidemiological data that contextualize Gal-1’s relevance across diverse populations and disease profiles [7,8]. Standardizing Gal-1 measurement methods and establishing global databases for clinical and experimental data would enhance research consistency and comparability. Drug development targeting Gal-1 presents both opportunities and challenges. While Gal-1 shows promise in modulating inflammation and vascular function, its complexity necessitates precise pharmacological interventions. Developing small-molecule inhibitors and monoclonal antibodies that selectively target Gal-1 without disrupting normal physiological functions is essential [58,59]. Additionally, nanoparticle-based delivery systems and RNA-based therapies could offer innovative strategies for localized Gal-1 modulation, reducing side effects and improving efficacy [60,69]. Investing in both basic and clinical research is critical to addressing gaps in our understanding of Gal-1 biology. Expanding clinical trials focused on Gal-1-targeted therapies for CVDs will help assess their safety, efficacy, and long-term outcomes. Further investigations into sex-specific cardiovascular differences and Gal-1’s link to metabolic diseases such as diabetes may provide deeper insights, guiding personalized treatment approaches [10,19]. By adopting these integrated and collaborative strategies, researchers can overcome existing barriers and unlock Gal-1’s full therapeutic potential in cardiovascular diseases, ultimately improving patient outcomes and advancing precision medicine.

Gal-1 presents significant therapeutic potential in CVDs, yet several obstacles hinder its clinical translation. These challenges include specificity in targeting, long-term safety concerns, and regulatory hurdles that affect both pharmacological and gene therapy approaches. Ensuring specificity in Gal-1-directed therapies remains difficult due to its structural similarities with other galectins, such as Gal-3 and Gal-9. Many inhibitors lack precision, leading to unintended immunological effects or disrupted glycan interactions [2,5]. Moreover, Gal-1 exhibits context-dependent effects, acting beneficially or detrimentally depending on concentration and disease progression [64]. This complexity necessitates the development of highly selective agents capable of fine-tuning Gal-1 activity while minimizing off-target interactions. Safety considerations further complicate the application of Gal-1 therapies. Because Gal-1 influences immune responses, fibrosis, and tumor progression, excessive inhibition could trigger unintended physiological disturbances [6]. Pharmacological inhibitors such as OTX008 and PTX008 have demonstrated promise in preclinical studies; however, limitations such as rapid systemic clearance and poor bioavailability pose challenges for effective therapeutic use [61,62]. Gene therapy approaches, although offering targeted intervention, introduce risks related to immune reactions, unintended genetic modifications, and difficulties in optimizing delivery [67]. Advances in nanoparticle-enhanced delivery methods and refined gene-editing technologies may mitigate these concerns and improve therapeutic precision [43]. Regulatory approval remains a significant hurdle for Gal-1-targeted therapies. While emerging evidence supports its therapeutic potential, extensive validation through large-scale clinical trials is necessary to confirm efficacy and safety [66]. Identifying appropriate patient populations and optimizing therapeutic windows are crucial steps before widespread adoption [60]. Pharmacological inhibitors must demonstrate consistent effectiveness across diverse demographics, ensuring minimal off-target effects [64]. Gene therapy approaches, given their long-term implications, require careful evaluation regarding stability, safety, and delivery standardization [15]. Advances in CRISPR-Cas9- and RNA-based modulation present promising avenues for precise Gal-1 regulation, though regulatory clearance will depend on comprehensive long-term safety assessments [65]. Despite these obstacles, continued advancements in Gal-1-targeted strategies hold potential for significant breakthroughs in cardiovascular therapeutics. Refining drug specificity, improving safety profiles, and addressing regulatory challenges through rigorous clinical trials will be essential in realizing Gal-1’s therapeutic viability. Integrating cutting-edge technologies such as gene-editing approaches and biomarker-driven precision medicine may further enhance targeting strategies, ultimately paving the way for optimized interventions in CVD management.

## 6. Conclusions

Gal-1 has emerged as a key factor in cardiovascular pathogenesis, exhibiting both protective and pathological effects depending on the stage of disease progression. Its involvement in inflammation, endothelial dysfunction, fibrosis, and immune modulation highlights its therapeutic potential across various cardiovascular conditions. While recent studies have provided valuable mechanistic insights into Gal-1’s actions, further research is needed to fully elucidate its regulatory mechanisms and explore its potential in mitigating cardiovascular diseases. The expanding body of evidence on Gal-1’s role in cardiovascular diseases has important implications for both research and clinical practice. Findings suggest its potential as both a biomarker for risk assessment and a target for therapeutic intervention [5,27]. Understanding the dual nature of Gal-1 and its influence on disease progression remains crucial for refining diagnostic and treatment strategies. Incorporating Gal-1 measurement into clinical practice could transform risk assessment and therapeutic approaches, enabling more personalized cardiovascular care. Despite challenges in fully characterizing its mechanisms and translating findings into clinical applications, Gal-1 continues to show significant potential for therapeutic development. Future research should focus on overcoming these barriers, addressing gaps in knowledge, and advancing targeted strategies to optimize Gal-1’s role in cardiovascular medicine. Ultimately, Gal-1’s integration into precision medicine approaches holds promise for improving patient outcomes and advancing cardiovascular healthcare worldwide.

## Figures and Tables

**Table 1:** Dual roles of galectin-1 in cardiovascular diseases.

Protective Effects of Gal-1	
Function	Protective Effects
Vascular Homeostasis	Promotes endothelial cell survival and angiogenesis, mitigating ischemic damage and supporting vascular integrity [1,27].
Inflammation Regulation	Suppresses pro-inflammatory cytokines (e.g., TNF-α, IL-6) and encourages M2 macrophage polarization, reducing chronic vascular inflammation [5,28].
Atherosclerosis Progression	Reduces expression of adhesion molecules (VCAM-1, ICAM-1), limiting monocyte infiltration and plaque formation [11,29].
Myocardial Protection	Alleviates ischemia–reperfusion injury by reducing cardiomyocyte apoptosis and oxidative stress, preserving cardiac function [30,31].
Heart Failure Modulation	Facilitates tissue repair and limits fibrotic remodeling, preserving myocardial function [19,32].
Calcium Channel Regulation	Modulates Ca(V)1.2 activity, reducing arterial constriction and preventing hypertension [21,22].
**Pathological Effects of Gal-1**	
**Function**	**Protective Effects**
Vascular Homeostasis	Disrupts endothelial function by increasing vascular permeability and driving inflammation, contributing to atherosclerosis and aneurysm progression [23,24].
Inflammation Regulation	Enhances leukocyte recruitment and activation, perpetuating vascular inflammation and worsening cardiovascular disease [33,34].
Atherosclerosis Progression	Promotes foam cell formation, lipid accumulation, and vascular smooth muscle cell migration, accelerating plaque instability [24,35].
Myocardial Protection	Contributes to maladaptive post-myocardial infarction remodeling and fibrosis, impairing cardiac performance [36,37].
Heart Failure Modulation	Induces fibrosis and excessive extracellular matrix deposition, leading to ventricular stiffening and diastolic dysfunction [38,39].
Calcium Channel Regulation	Disrupts calcium signaling in cardiomyocytes, contributing to contractile dysfunction and arrhythmias [9,32].

**Table 2:** Summary of human cohorts evaluating galectin-1 in cardiovascular diseases.

Disease Entity	Sample Size	Assay Type	Median/Mean Gal-1 Level (ng/mL)	Clinical Endpoints	Hazard Ratio (HR) (95% CI)
Critically Ill Patients	350	ELISA	Median: 39–70 (tertiles)	90-day mortality	HR: 3.21 (1.90–5.42) [25]
Acute Kidney Injury (AKI)	350	ELISA	Median: 39–70 (tertiles)	AKI within 48 h	HR: 2.88 (1.20–6.88) [25]
Coronary Artery Disease (CAD)	200	ELISA	Median: 56.3 (CAD) vs. 32.1 (controls)	Fibrosis, vascular stiffening	*p* < 0.01 [24]
Heart Failure (HF)	180	ELISA	Mean: 68.5 (HF) vs. 40.2 (non-HF)	Left ventricular diastolic dysfunction, hospitalization	HR: 2.45 (1.62–3.71) [37]

## Data Availability

As this is a review article, all data and materials referenced are drawn from publicly available studies and sources, as cited in the manuscript. Specific datasets, materials, or methodologies referenced in this review can be obtained from the original studies, which are listed in the References section. For any additional inquiries, please contact the corresponding author.

## Abbreviations

The following abbreviations are used in this manuscript:

Gal-1	Galectin-1
CVD	Cardiovascular diseases
HF	Heart failure
CAD	Coronary artery disease
HRs	Hazard ratios
CIs	Confidence intervals
MI	Myocardial infarction
BMSCs	Bone marrow stromal cells
CRD	Carbohydrate recognition domain
MMP	Matrix metalloproteinases
TIMPs	Tissue inhibitors of metalloproteinases
ECM	Extracellular matrix
TNF-α	Tumor necrosis factor-alpha
IL-6	Interleukin-6
IL-1β	Interleukin-1 beta
IL-10	Interleukin-10
VCAM-1	Vascular cell adhesion molecule-1
ICAM-1	Intercellular adhesion molecule-1
HFpEF	Heart failure with preserved ejection fraction
AAV	Adeno-associated viruses
